# Impaired Gastric Hormone Regulation of Osteoblasts and Lysyl Oxidase Drives Bone Disease in Diabetes Mellitus

**DOI:** 10.1002/jbm4.10212

**Published:** 2019-08-07

**Authors:** Eileen J Daley, Paola Divieti Pajevic, Sayon Roy, Philip C Trackman

**Affiliations:** ^1^ Boston University Henry M. Goldman School of Dental Medicine, Department of Molecular and Cell Biology Boston MA USA; ^2^ Boston University School of Medicine, Department of Medicine Boston MA USA

**Keywords:** COLLAGEN, BONE MATRIX, MOLECULAR PATHWAYS–REMODELING, BONE MODELING AND REMODELING, OSTEOPOROSIS, DISEASES AND DISORDERS OF/RELATED TO BONE, BONE QCT/μCT, ANALYSIS/QUANTITATION OF BONE, PRECLINICAL STUDIES, ANIMAL MODELS

## Abstract

Diabetic bone disease is a complication of type I and type II diabetes, both of which are increasing in the United States and elsewhere. Increased hip and foot fracture rates do not correlate well with changes in bone mineral density (BMD), whereas studies support the importance of collagen structure to bone strength. Extracellular lysyl oxidase (LOX) catalyzes the oxidative deamination of hydroxylysine and lysine residues in collagens resulting in aldehydes that subsequently form critically important biosynthetic crosslinks that stabilize functional collagens. Although LOX‐dependent biosynthetic crosslinks in bone collagen are deficient in diabetic bone, the expression and regulation of bone LOXs in diabetes have not been comprehensively studied. Here, we found that LOX is profoundly downregulated in bone in diabetes. Moreover, we have identified a novel metabolic regulatory relationship that is dysregulated in diabetes using mouse models. Data indicate that the incretin (gastric hormone) known as glucose‐dependent insulinotropic polypeptide (GIP) that is anabolic to osteoblasts strongly upregulates LOX, and that this regulation is disrupted in the streptozotocin‐induced model of diabetes in mice. In vivo and in vitro studies support that diabetes results in elevated circulating peripheral dopamine, likely also derived from the gut, and is responsible for blocking GIP signaling and LOX levels in osteoblasts. Moreover, peripheral administration of the dopamine D2 receptor antagonist amisulpride to diabetic mice restored trabecular bone structure to near normal and partially reversed downregulation of LOX. Taken together our data identifies a novel metabolic relationship between the gut‐derived hormone GIP and bone‐derived LOX, and points to the importance of LOX dysregulation in the pathology of diabetic bone disease. © 2019 The Authors. *JBMR Plus* published by Wiley Periodicals, Inc. on behalf of the American Society for Bone and Mineral Research.

## Introduction

The National Diabetes Statistics Report from the Centers for Disease Control and Prevention (CDC) in 2017 states that more than 100 million US adults are now living with diabetes, and it was reported as the seventh leading cause of death in the United States in 2015 (National Diabetes Statistics Report 2017; https://www.cdc.gov/diabetes/pdfs/data/statistics/national‐diabetes‐statistics‐report.pdf). Diabetes causes chronic issues in nearly every tissue, with extensive complications occurring in organs that are highly vascularized. Increased attention to connective tissue abnormalities associated with diabetes has identified bone fragility and increased susceptibility to fractures referred to as diabetic bone disease. Decreased bone health in diabetes has direct consequences of decreased mobility due to increased fracture risk that secondarily exacerbate the microvascular and macrovascular complications which can ultimately be fatal.^1^ The mechanisms of increased fracture risk are not entirely clear, but increased fracture risk in type II diabetes is often accompanied by increases (rather than decreases) in bone mineral density (BMD), pointing to the importance of the organic phase of bone to its structure and integrity.^1^, ^2^ The increased BMD in type II diabetes is likely due to increased bone formation as a result of mechanical loading, a consequence of obesity in type II diabetic patients.^3^ Diabetic bone loss in type I diabetes, however, is clearly associated with a low bone formation osteopenia, and type I diabetes animal models, therefore, are highly informative in the study of mechanisms of diabetic bone complications. Bone loss in type I diabetes is particularly severe compared to other metabolic bone diseases, with more than 50% of all patients and 20% of younger patients 20 to 56 years old meeting the criteria for being osteoporotic.^4^, ^5^

Low BMD is used to diagnose osteopenia but often does not correlate well with the actual impairment in mechanical properties seen in diabetic bone in type I and type II diabetes.^6^ Bone is a composite material consisting of both a mineral phase, composed of hydroxyapatite, and an organic phase that is mainly type I collagen. The amount and arrangement of both components of bone matrix determine the characteristics of bone. Previous studies have reported that in mouse models of diabetes, there is an abnormal arrangement of collagen fibrils that results in a significant reduction in bone material properties without affecting the mineral component of bone.^7^, ^8^ Results from these studies suggest that these collagen defects are caused by either accumulation of non‐enzymatic advanced glycation end products or the observed abnormally low levels of lysyl oxidase (LOX)‐mediated enzymatic crosslinks.^7^, ^9^

LOXs are copper‐dependent amine oxidases that oxidatively deaminate the epsilon amino group of peptidyl lysine and hydroxylysine residues to form reactive peptidyl aldehydes. These aldehydes react spontaneously with either each other or with unmodified lysine and hydroxylysine residues to form a variety of intramolecular and intermolecular crosslinks within and between collagen molecules.^10^ LOXs originate from five paralogous genes, lysyl oxidase (*LOX*) and LOX‐like 1 to LOX‐like 4 (*LOXL1–4*), which all contain the conserved C‐terminal enzyme domain. LOX family–dependent covalent crosslinking is required to stabilize collagen molecules and determines the strength and material properties of the collagen matrix of bone.^9^ Treatment of mice with β‐aminopropionitrile (BAPN), which inhibits all LOX isoforms, results in diminished crosslinking, and reduced bone volume, quality, and mechanical properties, including toughness, without affecting the mineral component. Toughness is the ability of a material to absorb energy and plastically deform without fracturing.^11^, ^12^ Therefore, low LOX activity negatively affects bone strength independent of changes in mineral properties, which mainly affect stiffness. Although stiffness is important for skeletal support, an ability to resist fracture requires both stiffness and toughness.^11^

The crosstalk between gastric hormones known as incretins and bone is a recently recognized contributor to normal skeletal metabolism. There are two hormones that act as incretins: glucose‐dependent insulinotropic peptide (GIP) and glucagon‐like peptide 1 (GLP‐1).^13^ GIP is directly anabolic to bone^14^, ^15^ and is secreted from intestinal K‐cells located in the distal duodenum in response to nutrient consumption, whereas GLP‐1 is not anabolic to bone.^16^ Incretins have multiple physiologic targets. When released after the ingestion of a meal, incretins are best known to bind to respective receptors on pancreatic β‐cells to stimulate insulin secretion. Incretins account for 50% to 70% of glucose control following a mixed meal, and underlie the mechanism by which oral glucose consumption results in a greater increase in insulin secretion than intravenous delivery of glucose. This phenomenon is known as the “incretin effect.”^17^ The human GIP receptor (GIPR) belongs to the seven‐transmembrane spanning heterotrimeric G‐protein coupled receptor family, and is expressed in pancreatic β‐cell islets and in adipose tissue, as well as in the heart, brain, and bone.^16^

GIPR is expressed on both osteoblasts and osteocytes, and GIP mediated signaling increases both alkaline phosphatase activity and collagen synthesis in osteoblasts, indicating GIP has anabolic activity independent of GIP‐stimulated insulin secretion.^18^ The binding of GIP to its receptor on osteoblasts increases cAMP production, indicating that GIP directly initiates intracellular signaling in bone‐forming cells.^19^ In GIPR^–/–^ global knockout mice, bone formation is decreased, accompanied by profound deficits in trabecular bone structure.^15^ In GIPR^–/–^ mice reduced crosslinking profiles correlate with reduced mechanical properties in long bones,^20^ suggesting that GIP is somehow needed to maintain the integrity of the collagen framework in bone.

Nutrient‐stimulated insulin secretion is deficient in diabetic patients. This has prompted investigation in to the effects of diabetes on incretin secretion and function. Serum GIP levels were found to be normal or slightly elevated in both type I and type II diabetes. GIP administration, even at higher than normal physiological doses, did not stimulate insulin secretion in diabetic humans,^21^ indicating that diabetes in some way interferes with GIP signaling. In diabetes, pancreatic β‐cells were still able to respond to GLP‐1, making GLP‐1 an attractive therapeutic for diabetes and leaving GIP to become known as the “forgotten incretin.”^13^ However GIP, not GLP‐1, has a direct effect on bone.

Bariatric surgery is an effective treatment for obesity and its associated comorbidities, including type II diabetes. Clinical follow‐up studies on obese patients who were also diabetic and had undergone bariatric surgery found that hyperglycemia was reversed in 83% of diabetic patients, and insulin secretion from the pancreas was significantly improved.^22^ Bariatric surgery commonly involves a duodenal jejunal bypass, which restricts nutrient access to the proximal small intestine by redirecting the stomach outlet to the distal intestine. Observations lead to the “foregut hypothesis,” which postulates that bypassing the upper small intestine restricts nutrient‐induced secretion of upper gastrointestinal factors that normally defend against hypoglycemia by reducing insulin secretion.^23^, ^24^ One potential gastrointestinal factor is gut‐derived dopamine, which in peripheral tissues is produced almost exclusively in the stomach and upper small intestine.^25^ Unlike its neurotransmitter counterpart in the brain, gut‐derived or peripheral dopamine travels through the peripheral bloodstream and does not cross the blood‐brain barrier. Studies on the function of gut‐derived dopamine are limited. Recent studies in rats revealed that increased peripheral dopamine production occurs after increases in GIP and insulin, followed by a decrease in circulating levels of GIP.^26^ This suggested the existence of a gut‐based mechanism for homeostatic glucose control, and provides circumstantial evidence that dopamine could represent an “anti‐incretin” capable of antagonizing incretin effects in the pancreas. This idea was extended in in vitro studies which revealed that treatment of pancreatic β‐cell islets with physiological concentrations of dopamine antagonized GIP‐stimulated increases in cytosolic cAMP and subsequent insulin secretion.^26^ Taken together these data suggest that gut‐derived dopamine could act as an anti‐incretin to prevent hypoglycemia in healthy individuals, and that this inhibitory circuit is in some way dysregulated in diabetes.

Here we present the hypothesis and supporting studies that the anti‐incretin hypothesis is relevant to diabetic bone complications. Dopamine receptors are expressed by osteoblasts^27^; however, the effect of dopamine on bone has only been studied in the context of dopamine as a neurotransmitter.^27^, ^28^ Interestingly, dopamine is produced primarily in the periphery, whereas only a minor fraction is synthesized in the brain.^25^ Here we propose a model for diabetic bone disease in which abnormally increased circulating gut‐derived dopamine in diabetes results in poor bone structure by antagonizing the GIP signaling in osteoblasts, leading to decreased LOX production and subsequent deficiencies in the organic matrix component of diabetic bone. Data presented here, performed in C57BL6 mice, osteoblasts, and MC3T3‐E1 cells, identify a novel gut/bone metabolic relationship that regulates osteoblast LOX that is dysregulated in diabetes, and importantly is amenable to therapeutic intervention.

## Materials and Methods

### Mouse models and tissues

All animal studies reported here were approved by the Boston University Institutional Care and Use Committee (protocol AN‐14302). Male and female mice were maintained in the Boston University Medical Center mouse facility with 12‐hour light and dark per day in cages of four mice each, and were fed normal mouse chow. Eight‐week‐old male and female C57BL/6 mice (The Jackson Laboratory, Bar Harbor, ME, USA) were made diabetic by the multiple low‐dose streptozotocin (MLD‐STZ)‐induction method, as we and others have described.^29^, ^30^ Daily i.p. injection to mice at 40 mg/kg doses of STZ (Sigma‐Aldrich, St. Louis, MO, USA; catalog# s0130) dissolved fresh in 0.1 M sodium citrate buffer solution, pH 4.5, were performed for 5 consecutive days. All mice were confirmed to be diabetic by 12 days after STZ initiation, evidenced by two consecutive fasting blood glucose readings of >250 mg/dL. Control mice were injected with vehicle only (sodium citrate buffer) and maintained normal blood glucose levels. Blood glucose levels were monitored twice a week for an additional 8 weeks, using a Onetouch Ultramini blood glucose meter and corresponding blood glucose test strips (LifeScan, Inc., Milpitas, CA, USA), which allows enough time for a diabetic bone phenotype to occur.^31^ Mice did not require insulin administration in these experiments, which used the MLD‐STZ model of diabetes induction.

To study the effects of dopamine on diabetic bone abnormalities, a subset of mice that had been diabetic for 8 weeks was then administered dopamine receptor inhibitor amisulpride for an additional 4 weeks. Ultra‐pure amisulpride (Sigma Aldrich; catalog# A2729) was first dissolved in sterile cell culture grade DMSO, and then dissolved in sterile PBS for a final concentration of 2% DMSO. Vehicle or amisulpride were administered by intraperitoneal injection at the same time every day for 4 weeks at a dosage of 10 mg/kg, which corresponds to a therapeutic dose used to treat schizophrenia in humans.^32^ An additional control group consisted of nondiabetic mice that were not injected with amisulpride. Amisulpride‐treated mice appeared to be slightly less active than normal, but no other behavioral effects were noted.

After sacrifice, the left femurs were dissected and wrapped in PBS‐soaked gauze for subsequent micro–computed tomography (μCT) analyses. The right femurs were fixed in 4% paraformaldehyde and then decalcified in 20% EDTA for 2 weeks. Femurs were then processed for histology using paraffin‐embedded sectioning by the Center for Skeletal Research histology core at Massachusetts General Hospital. The bones were embedded longitudinally with the intercondylar notch at the distal end facing upward.

### µCT and image analysis

All μCT analyses of femurs and L_5_ vertebrae were carried out at the µCT Imaging Core Facility at Boston University using a Scanco Medical µCT40 Scanning instrument (Scanco Medical, Brüttisellen, Switzerland). The power, current, and integration time used for all scans were 70 kVp, 113 μA, and 200 ms, respectively. The femurs and spines were all scanned at a resolution of 12 µm/voxel. The software used for the subsequent contouring and analysis was developed by Scanco Medical. Regions selected for trabecular analysis were the trabecular compartments of the distal metaphysis of the femur and the center of the vertebral body of the L_5_ vertebrae. For cortical analysis, the mid‐diaphysis of the femur and the cortical shell of the vertebrae were used for analysis.^33^ To determine the trabecular region of interest of the femurs, the location of the growth plate was determined and the regions that were 60 µm removed from the end of the growth plate and which extended 1200 µm proximal of the growth plate were respectively analyzed. The entire lengths of the vertebrae were evaluated, comprising a total length of ∼3600 µm. Gaussian filtering (sigma = 0.8, support = 1) was used for partial background noise suppression. The threshold was set at a 16‐bit grayscale value of 9830 and this global threshold was applied to all of the samples.^34^ For contouring the perimeter of the trabecular region of the vertebrae, each transverse 2D tomogram of the vertebra was manually traced to designate the cortical shell and trabecular regions. For separating the cortical and trabecular regions of the femurs, an automated method within the system software was used.^35^ Trabecular and cortical analyses were performed and reported as described.^33^

### Analysis of collagen organization

Collagen organization was analyzed using Picrosirius red–stained central sections as described.^36^ Picrosirius red stain was composed of 0.5 g Sirius Red dye (Direct Red 80; Sigma Aldrich; catalog# 43665) dissolved in 500 mL saturated aqueous picric acid. Sections were stained for 1 hour and counterstained with Weigert's hematoxylin. Images were visualized using an Olympus IX70 microscope with and without polarizing filters engaged (Olympus Inc., Tokyo, Japan) and captured using Pictureframe software. The area analyzed for each image started at the center of the growth plate and extended ∼2 mm down and to the right. Images were analyzed for blue, green, yellow, orange, and red birefringence. Images were taken at the same exposure and same orientation of the slide to ensure uniform birefringence.

### Isolation and analyses of RNA

Total RNA was extracted from the tibia of diabetic and nondiabetic mice, as well as amisulpride‐injected and vehicle‐injected diabetic and nondiabetic control mice. The tibia from each individual mouse was snap frozen in liquid nitrogen and ground to a fine powder using a mortar and pestle. The resulting powder was transferred to 1 mL of TRIzol extraction buffer, and RNA extracted using 0.2 mL chloroform. Samples were centrifuged, the aqueous phase removed, and further extracted using 1:1 volume of isopropyl alcohol. RNA was further purified using an RNeasy mini kit (QIAGEN, Valencia, CA, USA; catalog# 74004). cDNA was synthesized from 1 µg total RNA using TaqMan Reverse Transcription Reagents (Applied Biosystems, Foster City, CA, USA; catalog# N8080234). cDNAs were then subjected to real‐time PCR analysis (TaqMan gene expression) using Applied Biosystems 7300 Real‐Time PCR systems to measure LOX mRNA levels using TaqMan probes from Applied Biosystems: mouse LOX (Mm00495386_m1) and GAPDH internal control (Hs02786624_g1). Results were calculated using the –2^ΔCT^ or 2^–ΔΔCT^ quantification methods, and plots generated using Graph Pad software (GraphPad Software, Inc., La Jolla, CA, USA).

### Serum immunoassays

Serum samples from nondiabetic and diabetic mice were analyzed for GIP (Mybiosource, San Diego, CA, USA; catalog# MBS268941) dopamine (Mybiosource; catalog# MBS732020), and insulin (Thermo Fisher Scientific, Rockford, IL, USA; catalog# EMINS) using ELISA assays in accordance with the manufacturer.

### Cell culture and analysis of GIP‐mediated LOX regulation

For signaling studies to elucidate the GIP signaling pathway in osteoblasts, the calvaria preosteoblast cell line MC3T3‐E1 was used (American Type Culture Collection [ATCC], Manassas, VA, USA; CRL‐2593). Cells were cultured in α‐MEM medium supplemented with 10% FBS and 1% penicillin/streptomycin. Cells were plated in six‐well plates at a density of 1 × 10^6^ cells per well. Cells were serum‐depleted overnight and then treated with 0 nM, 1 nM, and 10 nM GIP (Tocris Bioscience, Bristol, UK; catalog# 2084) as well as 10 μM forskolin, an adenylyl cyclase agonist. Cells were also pretreated with 100 µM of the adenylyl cyclase inhibitor SQ22536 (Tocris Bioscience; catalog# 1435) and the myristoylated PKA inhibitor PKI‐22 (Enzo Life Sciences, Farmingdale, NY, USA; BML‐P210‐0500) for 30 min before treatment with either GIP or forskolin. RNA was extracted using the RNeasy mini kit and subjected to RT‐qPCR using the same LOX gene TaqMan probe described in "Isolation and Analysis of RNA". For absolute quantification, data were calculated based on a standard curve of known transcript copy numbers and normalized to the amount of cDNA added. LOX cDNA was serially diluted to known concentrations and a standard curve was generated where concentration was plotted against C_q_ quantification cycle values. C_q_ values from unknown samples were obtained by qPCR and concentration was extrapolated from the standard curve and converted to copy number.

Protein was extracted by lysing cells directly in protein sample buffer (0.5 M Tris, 10% glycerol, 2.5% SDS) and lysates were subjected to Western blotting to measure the levels of LOX protein. Medium was also taken from these same cells, and concentrated using an Amicon Ultra 10 K filter (Amicon, Millipore, Billerica, MA, USA; catalog# UFC801096). For Western blotting, samples were separated on 10% SDS‐PAGE gels and transferred overnight to a polyvinylidene fluoride (PVDF) membrane. Membranes were blocked for 2 hours in 5% nonfat dry milk in TBS‐T and then incubated with LOX antibody specific for the C‐terminal domain of LOX, which is conserved in humans and mice at a dilution of 1:1000 (Abcam, Cambridge, MA, USA; catalog# ab31238). Membranes were then washed with TBS‐T and incubated with anti‐rabbit horseradish peroxidase (HRP)‐coupled IgG at a 1:2000 dilution. Membranes were washed again and visualized with HyGlo Quick spray (Denville Scientific, Holliston, MA, USA; catalog# E2400), and imaged using the SynGene GBox and Genesys software (SynGene, Frederick, MD, USA). The membranes were then stripped using Restore Western Stripping Solution (Pierce, Rockford, IL, USA) and re‐probed with anti‐β‐actin antibody (Cell Signaling Technology, Beverly, MA, USA; antibody #4967) as a loading control.

### cAMP assay

MC3T3‐E1 osteoblasts were plated in 96‐well plates, serum starved overnight, and treated with 0 nM, 0.1 nM, 1 nM, and 10 nM GIP, as well as 10 µM forskolin, for 4 hours in the presence of 10 μM 3‐isobutyl‐1‐methylxanthine (IBMX), a phosphodiesterase inhibitor. Cells were also pretreated with 100 μM of the adenylyl cyclase inhibitor SQ22536 for 30 min before treatment with either GIP or forskolin. Cells were lysed in 6 N HCl and subjected to a radioimmunoassay for cAMP. Assays were performed by the cell signaling core at the Center for Skeletal Research at Massachusetts General Hospital. Conditions were run in triplicate and the assay was performed three times.

### Analysis of PKA activity

MC3T3 cells were plated in six‐well plates, serum‐depleted overnight, and treated with 0 nM, 0.1 nM, 1 nM, 10 nM, 100 nM, 200 nM, and 1 µM GIP, as well as forskolin for 1 hour, while some cells were pretreated for 30 min with the myristoylated PKA inhibitor PKI‐22, followed by GIP treatment. Cells were lysed directly in SDS‐PAGE sample buffer and lysates were subjected to Western blotting to measure the levels of phosphorylated cAMP response element‐binding protein (phospo‐CREB) and total CREB. Blocked PVDF membranes were incubated with an antibody specific for phosphorylation site Ser133 on CREB (Cell Signaling Technology; rabbit mAb #9198) and imaged using the SynGene GBox and Genesys software. The membranes were then stripped and probed with anti‐total CREB antibody (Cell Signaling Technology; rabbit mAb #9197) to normalize phospho‐CREB levels. Densitometry analysis was carried out using the gel analyzer feature of the Image J software according to the user manual (IJ version 1.46r, section 30.13). The phospho‐CREB levels were reported as a ratio of phospho‐CREB band to total CREB band intensity levels.

### Dopamine inhibition studies

MC3T3‐E1 osteoblasts were plated in 96‐well and six‐well plates. Cells were pretreated with dopamine hydrochloride (Sigma Aldrich; catalog# H8502) at concentrations of 0 nM, 10 nM, 100 nM, 1 µM, 10 µM, and 100 µM for 30 min before treatment with 10 nM GIP for 4 hours. cAMP assays and Western blotting for LOX protein levels were then performed. Primary bone cells isolated from nondiabetic and diabetic mice were also plated in 96‐well and six‐well plates. Cells were pretreated with the dopamine receptor inhibitor amisulpride at a concentration of 20 μM for 30 min before treatment with GIP for 4 hours. cAMP assays and qRT‐PCR for LOX mRNA levels were then performed.

### Primary cell isolation

To study the effects of diabetes on osteoblasts, primary osteoblasts were isolated from the right and left tibia of diabetic and nondiabetic control mice.^37^ The Boston University Institutional Animal Care and Use Committee (IACUC) approved all animal protocols. The clean diaphysis were cut into pieces of approximately 1 to 2 mm^2^ using scissors and fragments were placed in a 5‐mL culture flask. The bone pieces were then washed several times with PBS and incubated in 4 mL collagenase II solution (2 mg collagenase II powder (Thermo Fisher Scientific; catalog# 17101015) per mL DMEM, made fresh and filter sterilized) at 37°C on a shaking platform in order to remove all remaining soft tissue and adherent cells. After 2 hours, the bone pieces were rinsed three times with DMEM supplemented with 100 µg/mL ascorbate and 10% FBS that was made fresh before each medium change and filter sterilized. The bone pieces were then transferred to 25‐cm^2^ flasks, containing 5 mL medium, at a density of about 20 fragments per flask. Adult mouse bone cells started to migrate from the bone chips after about 2 days. On day 4, cells and medium were harvested for protein and RNA isolation, or cells were passaged by incubation with 0.25% trypsin for 10 min, and then replated at a density of 2.5 × 10^3^ to 5 × 10^3^ cells per cm^2^ for signaling experiments. For differentiation experiments, cells were grown to approximately 60% to 70% confluence and growth medium was replaced with osteogenic differentiation medium (α‐MEM containing freshly prepared ascorbic acid [50 μg/mL], β‐glycerophosphate [10 mM], and dexamethasone [10 nM]). During the course of differentiation the media were refreshed every 3 days.

Experiments with differentiated mouse primary osteoblasts were conducted on day 14 and day 21. Total RNA and protein was extracted by direct lysis of diabetic and nondiabetic primary cells. RNA was also isolated from differentiated diabetic and nondiabetic primary cells and subjected to RT‐qPCR. TaqMan probes used are as follows: Osterix (SP7) (Mm04933803_m1), RUNX2 (Mm00501584_m1), Osteocalcin (BGLAP)(Mm03413826_mH), BSP(IBSP) (Mm00492555_m1), and DMP‐1(Mm01208363_m1).

### Statistical analysis

All signaling experiments were run in triplicate and repeated three times. For in vivo experiments, *n* = 8 or 10, as indicated. One‐way ANOVA and Tukey's post hoc test for specific differences in between groups was employed for all analyses involving multiple groups, and Student's *t* test employed for all other analyses. Significance was declared as *p* < 0.05. Data are presented as means ± SD.

## Results

### Diabetic mice have decreased trabecular bone quality and altered collagen structure

**Figure 1:**
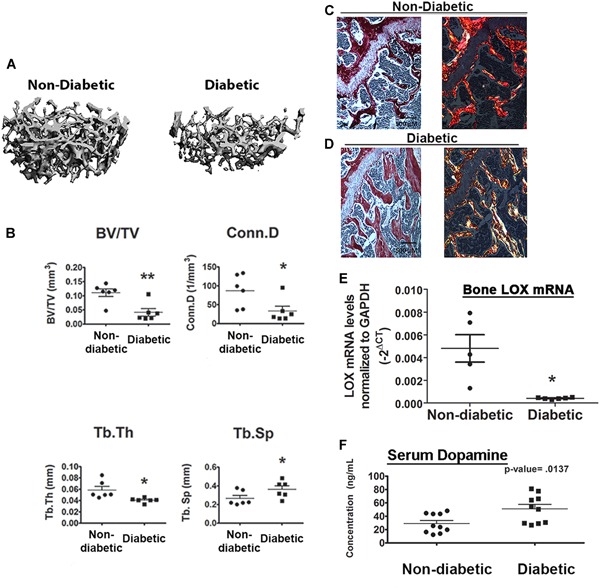
Bone phenotype of nondiabetic and diabetic mice. µCT 3D tomogram (*A*) and trabecular bone parameters (*B*) from distal metaphysis of the femur in nondiabetic and diabetic mice. Asterisks (*) are versus wild‐type; *p < 0.05; **p < 0.01, *n* = 6 by Student's *t* test. (*C*, *D*) Collagen is disrupted in diabetic bone. Collagen profile analysis of nondiabetic and diabetic mice. Picrosirius red staining of collagen under nonpolarized (left panel) and polarized light (right panel) in the femur. Representative images are shown, *n* = 6 with three sections per animal. (*E*) Diabetes reduces LOX mRNA in bone. Relative LOX mRNA in nondiabetic and diabetic mouse femurs in vivo. RNA was isolated directly from right femurs. Data were calculated using the –2^–(ΔCT)^ method, and statistics analyzed by Student's *t* test with Welch's correction for unequal variances, *p = 0.022 in GraphPad. (*F*) Serum dopamine is elevated in diabetic mice. Serum dopamine levels in nondiabetic and diabetic mice. Concentration of dopamine (ng/mL) was increased in diabetic mice. Data are means ± SD, *p < 0.05, by Student's *t* test, *n* = 10 per group.

A subset of C57Bl/6 wild‐type male and female mice was made diabetic by low‐dose streptozotocin‐induction; all became diabetic by 12 days (>250 mg/dL serum glucose) (*n* = 8) and remained diabetic for an additional 8 weeks, while control mice remained nondiabetic. We first examined if these diabetic mice were osteopenic and could recapitulate the specific bone abnormalities observed in the metabolically active trabecular bone regions, and in the appendicular as opposed to the axial skeleton.^5^, ^6^, ^38^ Mice were euthanized 8 weeks after diabetes induction; bones were dissected and scanned using a Scanco Medical µCT40 Scanning instrument. Diabetic mice showed defects in trabecular structure in the femur, which can be seen in the 3D tomogram (Fig. 1 *A*). Quantitation of data according to established parameters^33^ showed that the bone volume/total volume (BV/TV) and connectivity density (Conn.D) were reduced to 20% of control levels, and trabecular thickness (Tb.Th) to half of control levels, whereas trabecular spacing (Tb.Sp) was significantly increased by 50% compared to nondiabetic control mice (Fig. 1 *B*). These data indicate there was less total trabecular bone in diabetic mice. There were no significant differences in cortical BV/TV in the diabetic mice model (Supporting Fig. 1), consistent with previous studies.^5^ Examination of the L_5_ vertebrae also showed that diabetic mice had defects in the trabecula of the vertebrae (Supporting Fig. 2). However, the phenotype was not as severe as that seen in the femur, with significant differences only seen in the BV/TV and Tb.Sp (Supporting Fig. 2 *B*).

We next assessed for defects in the collagen structure of diabetic mouse bones directly by Picrosirius red histology. In the nonpolarized light panel, the amount of collagen appeared similar between nondiabetic and diabetic mice (Fig. 1 *C*, *D*). However, under polarized light the diabetic mouse bones had a very different birefringence profile than that of nondiabetic mice (Fig. 1 *C*, *D*). In Picrosirius red histology, the picric acid can more easily dissolve collagen fibers that are loosely packed. When picric acid is able to penetrate the collagen, a bright yellow birefringence is observed under polarized light (Fig. 1 *D*). When the stain is less able to penetrate the collagen due to tight fibril packing, the collagen profile, as seen in the nondiabetic mice, appears less bright under polarized light, with birefringence mixed with evidence of green, yellow, blue, and some orange red, and yellow.

**Figure 2:**
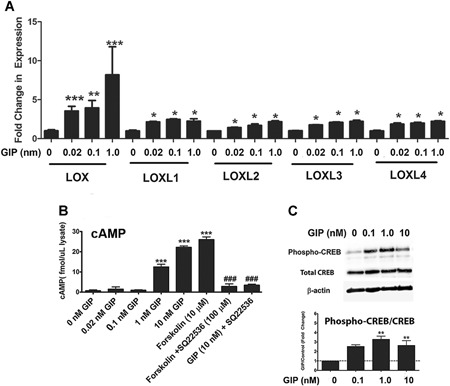
LOX paralogs regulation by GIP (*A*) MC3T3‐E1 cells were treated for 24 hours with a range of GIP concentrations followed by RNA isolation and qPCR. Data were calculated using the 2^–(ΔΔCT)^ method and are provided as fold change relative to GAPDH. Data are means ± SD, *p < 0.05, **p < 0.01, ***p < 0.001 from untreated by one‐way ANOVA with Tukey's multiple comparisons test. (*B*) In vitro cAMP accumulation in response to GIP treatment. MC3T3 osteoblasts were treated with a range of GIP concentrations for 4 hours in the presence of IBMX (phosphodiesterase inhibitor) and SQ22536 (adenylyl cyclase inhibitor) or Forskolin (adenylyl cyclase activator) as indicated. Data are means ± SE, *p < 0.05, **p < 0.01, ***p < 0.001 indicate difference from untreated, while #*p* < 0.05, ##*p* < 0.01, and ###*p* < 0.001 indicate difference from 10 nM GIP by one‐way ANOVA and Tukey's multiple comparisons test. (*C*) GIP activation of CREB activating phosphorylation. MC3T3 cells were treated with 10 nM GIP for 15 min. Cell lysates were probed with antibodies recognizing PKA phosphorylation site Ser133 on CREB and total endogenous CREB, and β‐actin. This experiment was repeated three times and data are means ± SE, **p < 0.01, indicating differences from untreated cells by one‐way ANOVA and Tukey's multiple comparisons test; asterisks above the bars indicate differences between groups.

### Diabetic mice have decreased bone LOX expression

LOX is required for collagen crosslinking, and the bone phenotype seen by Picrosirius red staining indicated aberrant collagen organization and structure, suggesting a possible role for LOX. LOX mRNA levels in diabetic bone have never been measured before, to our knowledge. Therefore, we sought to investigate our hypothesis that LOX is dysregulated in diabetic bone by examining levels of LOX in both nondiabetic and diabetic mice. RNA was isolated directly from the tibia of the nondiabetic and diabetic mice and the expression of LOX was analyzed by qRT‐PCR. Results show that LOX mRNA levels in diabetic bone were reduced to 8% of levels observed in nondiabetic bones (Fig. 1 *E*), suggesting that LOX downregulation could play a critical role in diabetic bone disease.

### Diabetic mice have increased serum dopamine levels

After we had established that diabetic mice had decreased LOX levels, aberrant collagen structure, and decreased trabecular bone volume and defective trabecular structure, we next sought to investigate specific molecular mechanisms occurring in diabetes that could result in these effects on bone. We hypothesized that increased gut‐derived dopamine in diabetes could cause diabetic bone abnormalities by antagonizing the GIP signaling pathway in osteoblasts. This hypothesis is based on temporal relationships between gut‐derived GIP and dopamine secretion, and inhibition of GIP signaling by dopamine in pancreatic β‐cells,^25^, ^26^ as noted in the Introduction. Serum dopamine levels increased significantly in mice 8 weeks after the onset of diabetes compared to the nondiabetic controls (Fig. 1 *F*), supporting the hypothesis that increased peripheral dopamine could contribute to the diabetic bone phenotype seen in these mice. Of note, an ELISA for GIP on serum from diabetic and nondiabetic mice shows GIP levels did not decrease in diabetic mice compared to nondiabetic control mice (Supporting Fig. 3), confirming the results of previous studies.^21^, ^39^

**Figure 3:**
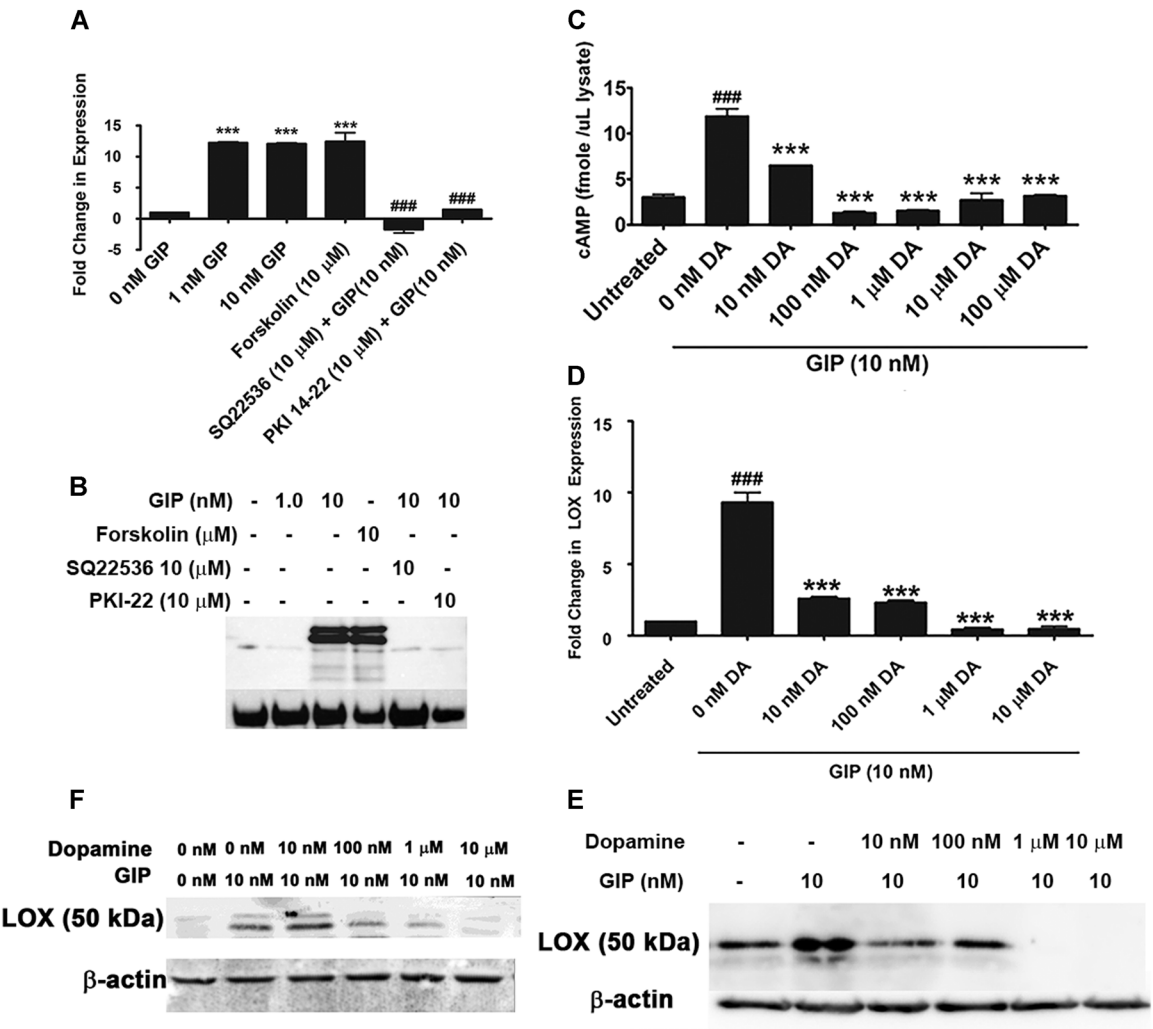
(*A*) LOX mRNA regulation by GIP depends on PKA. MC3T3 cells were treated with GIP for 4 hours in the presence and absence of PKA pathway activators and inhibitors, and RNA was analyzed for LOX levels. LOX RNA levels were increased by GIP, which was prevented when cells were treated with the adenylyl cyclase inhibitor SQ22536 or the PKA inhibitor PKI 14‐22. Data were calculated using the 2^–(ΔΔCT)^ method and are represented as fold change relative to GAPDH. Data are means ± SE, ***p < 0.001 indicate difference from untreated, while ###*p* < 0.001 indicates difference from 10 nM GIP (by one‐way ANOVA with Tukey's multiple comparison test). (*B*) GIP regulation of LOX protein. Western blot for pro‐LOX protein expression after GIP treatment. MC3T3 cells were treated with GIP for 4 hours and LOX cell layer protein expression was determined. Cells were also pretreated with the adenylyl cyclase inhibitor SQ22536 and the PKA inhibitor PKI 14‐22. β‐actin was employed as loading control. DA inhibits GIP stimulated (*C*) cAMP increases and (*D*) LOX mRNA levels. Treatment of MC3T3 cells with 10 nM GIP for 4 hours results in an increase in cytosolic cAMP levels and LOX mRNA. Pretreatment for 30 min with 10 µm to 100 µM DA blocks this increase, suggesting DA is able to antagonize GIP stimulated cAMP and LOX RNA production from adenylyl cyclase. In *C* and *D*, data are means ± SD, ***p < 0.001 compared to 0 nM DA treated with GIP; ###*p* < 0.001 difference from untreated cells by one‐way ANOVA and Tukey's test for multiple comparisons. (*E*, *F*) DA inhibits GIP‐stimulated LOX protein levels. Western blot of cell extracts for LOX after GIP treatment ± DA of (*E*) MC3T3 cells and (*F*) primary osteoblasts for 4 hours, with or without pretreatment for 30 minutes with various concentrations of DA. Cells were grown to 80% visual confluence, plated in six‐well plates, and were serum starved for 18 hours prior to treatment. Cell layers were extracted directly in to sample buffer and Western blots were probed for total LOX protein. DA = dopamine.

### GIP upregulates osteoblast LOX by an adenylyl cyclase/cAMP/PKA‐dependent pathway

Although previous studies have shown that GIP causes an increase in bone formation and LOX‐dependent collagen crosslinking in osteoblasts,^14^, ^19^ the specific mechanisms by which GIP upregulates LOX have not been investigated. We therefore sought to determine the downstream effectors in this pathway under normal conditions to better understand what is affected under diabetic conditions.

To investigate whether GIP upregulates LOX mRNAs and to determine which paralog is predominantly affected, MC3T3‐E1 calvaria osteoblast cells were treated with GIP at concentrations ranging from 0.02 nM to 1 nM. Relative mRNA expression of the LOX paralogs normalized to a GAPDH internal control was determined by qRT‐PCR for all five paralogs of LOX. This was important to determine because each paralog is regulated independently.^10^, ^40^ Data showed that LOX was upregulated more than eightfold at 1 nM GIP in MC3T3‐E1 osteoblasts after 24 hours, whereas the other LOX isoforms exhibited lower degrees of upregulation of about twofold (Fig. 2 *A*). This finding provides the first direct evidence that GIP strongly increases LOX expression, and supports the hypothesis that loss of GIP regulation of LOX in diabetes could contribute to decreased collagen crosslinking and osteopenia observed in diabetes.

The receptor for GIP (GIPR) is a class II G‐protein coupled receptor (GPCR) whose activity is mediated mainly through the Gs_α_ protein subunit,^41^ possibly upstream of the adenylyl cyclase/cAMP/PKA pathway.^42^, ^43^ Previous studies have shown that GIP signaling is dependent on the production of cAMP by adenylyl cyclase in pancreatic β‐cells.^16^ However, inhibitors of PKA do not completely abrogate the effects of GIP on insulin secretion in β‐cells,^44^ suggesting the potential involvement of additional noncanonical pathways activated by the GIP receptor. To evaluate the extent of cAMP accumulation in response to GIP, MC3T3‐E1 cells were treated with GIP at concentrations ranging from 0.1 nM to 10 nM for 4 hours. The adenylyl cyclase agonist forskolin or the adenylyl cyclase inhibitor SQ2256 were used as controls. Data show that treatment of MC3T3‐E1 cells with GIP increased cAMP levels starting at concentrations of 0.1 nM, with a response of more than 20 fmol/µL cell lysate occurring at concentrations of 10 nM GIP after 4 hours (Fig. 2 *B*). Forskolin had similar effects, whereas pretreatment of the cells with the adenylyl cyclase inhibitor SQ22536 significantly inhibited both GIP‐induced and forskolin‐induced cAMP responses. These data confirm that GIPR signaling stimulates adenylyl cyclase. To determine if GIP caused an increase in PKA activity, the phosphorylation of the downstream targets of PKA were determined by Western blot as a measure of kinase activity. PKA activating phosphorylation of its major substrate CREB was determined using phospho‐CREB antibodies normalized to total endogenous CREB. GIP caused an increase in the ratio of phosphorylated to total CREB in MC3T3‐E1 osteoblasts (Fig. 2 *C*), suggesting PKA enzymatic activity was increased in response to GIP in osteoblasts.

To investigate the direct involvement of both cAMP and PKA in LOX regulation, differences in levels of LOX were examined at the RNA and protein level. Cells were pretreated with forskolin, the adenylyl cyclase inhibitor SQ2256, or the myristoylated PKA inhibitor PKI‐22 followed by 10 nM GIP. qRT‐PCR and Western blotting analysis of cell extracts showed that the increase in cAMP and PKA enzymatic activity in response to GIP is accompanied by an increase in LOX mRNA (Fig. 3 *A*) and protein levels (Fig. 3 *B*). The two bands on the Western blot for LOX correspond to N‐glycosylated non‐glycosylated pro‐LOX during its endoplasmic reticulum (ER) processing.^45^ Pretreatment with both the adenylyl cyclase or PKA inhibitor dramatically inhibited GIP upregulation of LOX at both the RNA and protein levels (Fig. 3 *A*, *B*), indicating that GIP‐stimulated increases in LOX expression in osteoblasts is dependent on both cAMP production by adenylyl cyclase and PKA activation.

### Dopamine inhibits GIP‐stimulated increases in cAMP, LOX mRNA, and protein production in osteoblasts

In pancreatic β‐cells, the dopamine receptor 2 (D2R) is the predominant receptor expressed and couples to G‐protein complexes containing Gαi_/o_ proteins that inhibit adenylyl cyclase, which antagonizes GIP stimulated increase in cAMP.^26^ Because osteoblasts also express D2R,^27^ we next determined if a similar mechanism occurs in osteoblasts. We therefore performed radioimmunoassay (RIA) cAMP assays in MC3T3‐E1 osteoblasts and this time pretreated for 30 min with physiologically relevant concentrations of dopamine.^26^ Results showed that 10 nM dopamine and optimal at 100 nM dopamine pretreatment significantly inhibited 10 nM GIP‐stimulated increases in cAMP in MC3T3‐E1 osteoblasts (Fig. 3 *C*, *p* < 0.001), with cAMP levels at or below baseline levels in untreated cells. These results indicate that dopamine at physiologically relevant concentrations is able to inhibit GIP signaling in osteoblasts.

To investigate if dopamine promotes diabetic bone disease by inhibiting LOX in osteoblasts, we next examined the ability of dopamine to inhibit the GIP‐stimulated LOX protein production. MC3T3‐E1 and primary osteoblasts were pretreated with the same concentrations of dopamine for 30 min before adding 10 nM GIP. We then isolated RNA and protein from cell lysates and subjected them to qRT‐PCR and Western blotting for LOX protein. Results demonstrated that pretreatment with 10 nM dopamine inhibited GIP‐stimulated increases in osteoblast LOX mRNA (Fig. 3 *D*) and protein production (Fig. 3 *E*, MC3T3 cells; Fig. 3 *F*, primary mouse osteoblasts), with complete inhibition of LOX occurring at 1 µM dopamine at both the RNA and protein levels. These results show for the first time that dopamine inhibits LOX production in osteoblasts, which is essential for normal bone formation. These results combined with data showing increased peripheral dopamine in diabetes support our working hypothesis.

### Diabetic primary bone cells produce low amounts of LOX mRNA and protein

**Figure 4:**
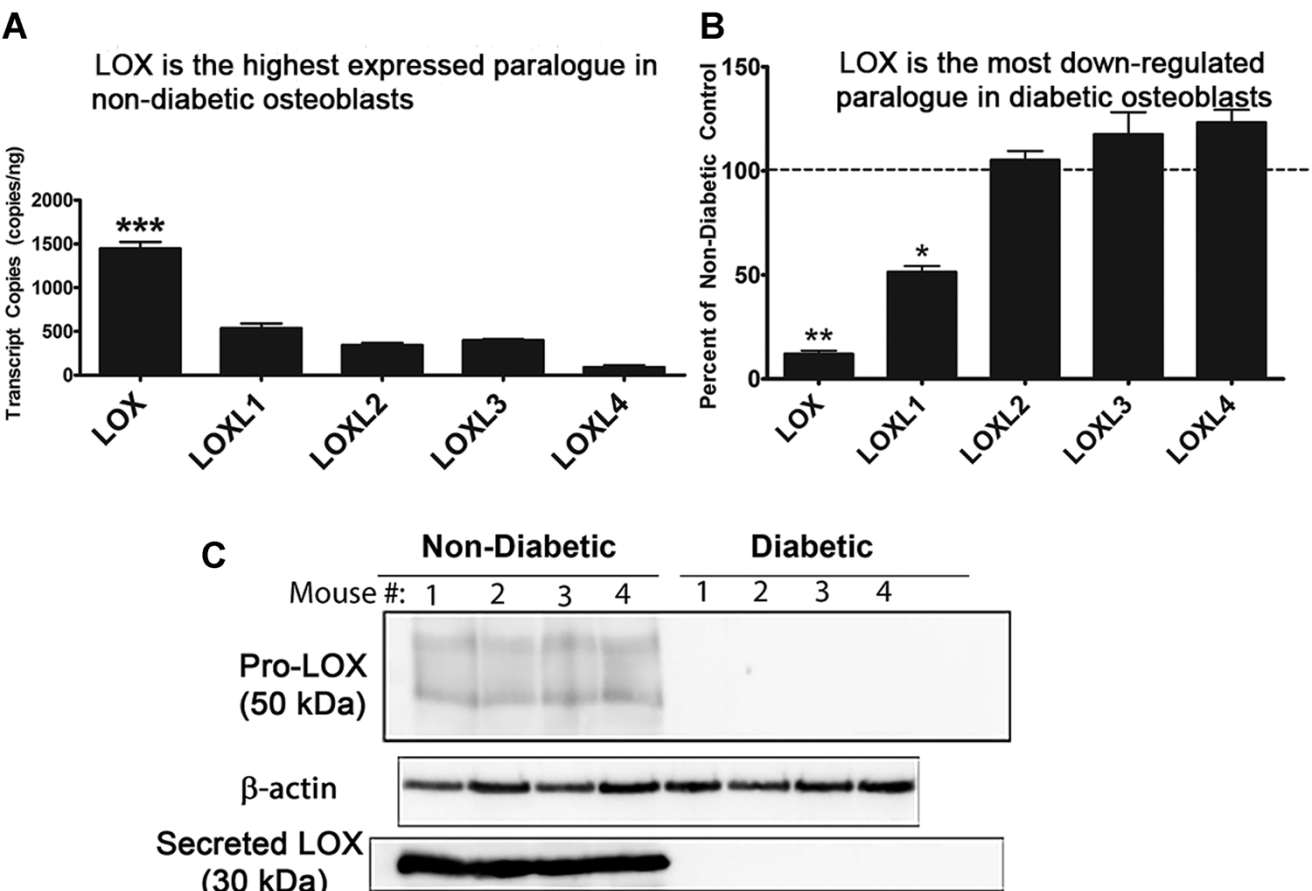
Diabetic primary bone cells. (*A*) LOX is the highest expressed paralog in nondiabetic primary osteoblasts. Absolute quantification of LOX isoforms in nondiabetic primary osteoblasts. Data were calculated based on a standard curve of known transcript copy numbers and normalized to the amount of cDNA added (*n* = 5). (*B*) LOX is the most highly inhibited paralog in primary diabetic osteoblasts. Data represent percent of nondiabetic controls calculated using the ΔΔCT method using GAPDH internal control. Data are pooled from three independent experiments (data are means ± SE, *p < 0.05, **p < 0.01, ***p < 0.001 by one‐way ANOVA and Tukey's test for multiple comparisons, *n* = 5 per group (diabetic and nondiabetic). (*C*) Cell layer and secreted LOX protein levels are decreased in primary bone cell cultures. Primary bone cells were plated at density of 5 × 10^3^ cells per cm^2^ in 10‐cm plates, lysed after 4 days, and cell lysates were probed for pro‐LOX and β‐actin via Western blot. Medium from the same plates was concentrated 40 × and equal volumes assessed for mature LOX protein. *n* = 4 per group with each lane representing a respectively numbered mouse.

Primary bone cells were isolated from the femurs of diabetic and nondiabetic mice to further investigate the anti‐incretin/LOX hypothesis (*n* = 5). First, primary bone cells from both groups of mice were differentiated using osteogenic differentiation medium to confirm that the primary bone cell isolation was successful. RNA was isolated from diabetic and nondiabetic primary bone cells on day zero and on day 21 after initiating differentiation medium as indicated in the Materials and Methods, and subjected to qRT‐PCR analysis for osteoblast differentiation markers osterix, RUNX2, osteocalcin, bone sialoprotein (BSP), and late‐stage osteoblast differentiation marker dentin matrix protein‐1 (DMP1). Both nondiabetic and diabetic bone cells were able to similarly differentiate into osteoblasts that express high levels of osteocalcin, BSP, and DMP‐1 (Supporting Fig. 4), indicating that the cells isolated were in fact mainly osteoblasts. We also confirmed that these osteoblasts express mRNA transcripts for LOX and the LOX isoforms LOXL1 to LOXL4 using absolute quantification qRT‐PCR on RNA isolated from primary bone cells after 4 days after culture, with the idea that they would still maintain their diabetic profile with a short culture time. Data indicated that LOX was the predominant isoform expressed in the nondiabetic primary osteoblasts, followed by LOXL1 (Fig. 4 *A*), consistent with data published by others.^46^ We then performed relative quantification of each isoform from qRT‐PCR data in the diabetic versus nondiabetic cells using GAPDH as internal control. Results show that diabetic primary bone cells showed a 90% reduction in LOX expression, whereas LOXL1 was reduced by 50% and LOXL2 to LOXL4 were unaffected (Figure 4B). We isolated protein from nondiabetic and diabetic primary bone cells after 4 days in culture and examined LOX protein levels by Western blot in both the cell lysate as a measure of intracellular LOX, and in the concentrated medium as a measure of secreted LOX. Data show that diabetic primary bone cells produced less intracellular pro‐LOX protein compared to control nondiabetic primary bone cells (Fig. 4 *C*, top panel), corroborating the qRT‐PCR results and showing both transcriptional and translational defects in LOX production. Diabetic primary bone cells did not secrete detectable LOX in to the medium, whereas nondiabetic primary bone cells did (Fig. 4 *C*, bottom panel). These defects in LOX production and secretion by primary bone cells isolated from diabetic mice are fully consistent with the defects in collagen structure and trabecular abnormalities observed in the skeleton of these mice.

### Inhibiting D2R signaling restores GIP‐stimulated cAMP production and LOX expression in diabetic primary bone cells

**Figure 5:**
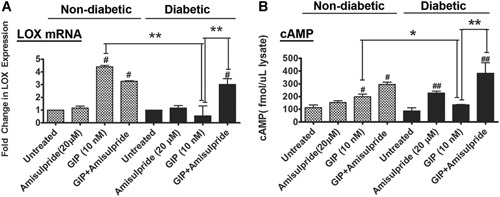
D2R inhibitor amisulpride restores GIP‐stimulated cAMP production and LOX expression in diabetic primary bone cells. (*A*) RT‐PCR for LOX RNA transcripts from primary bone cells isolated from diabetic and nondiabetic mice as a function of GIP and D2R inhibitor treatment and (*B*) Analysis of cAMP production. Primary osteoblasts pretreated for 30 min with or without 20 µM D2R inhibitor amisulpride followed by 10 nM GIP. Data are means ± SD, #*p* < 0.01, ##*p* < 0.005, difference from untreated nondiabetic or diabetic control, *p < 0.05, **p < 0.01, difference between groups by one‐way ANOVA with Tukey's test for multiple comparisons. Data are from pooled primary cells from five diabetic and five nondiabetic mice.

We next determined if diabetic bone abnormalities could be treated by rescuing GIP signaling and LOX production in primary osteoblasts isolated from diabetic mice using amisulpride, which is a selective inhibitor of the dopamine receptor D2R.^32^ Primary cells isolated from diabetic and nondiabetic control mice were cultured and pretreated for 30 min with 20 µM amisulpride followed by 10 nM GIP. We wanted to determine (i) if primary bone cells isolated from diabetic mice were able to respond to GIP in the same manner as the nondiabetic primary bone cells; and (ii) if they were unable to respond, if we could restore the GIP response by inhibiting signaling through the D2R. LOX mRNA levels by qRT‐PCR analysis of cell lysates in the presence and absence of amisulpride was performed, and cAMP analysis was performed as a readout for GIP receptor signaling. Results in cells from diabetic mice (*n* = 5), interestingly, showed that amisulpride restored GIP‐stimulated LOX mRNA levels to GIP‐stimulated wild‐type levels (Fig. 5 *A*), and similarly recovered GIP‐stimulated cAMP increases in cells from diabetic mice (Fig. 5 *B*). GIP treatment alone in the primary bone cells isolated from nondiabetic mice stimulated an increase in cAMP as expected, but failed to do so in primary bone cells isolated from diabetic mice (Fig. 5 *B*, *p* < 0.05). Treatment of diabetic osteoblasts with amisulpride alone (Fig. 5 *B*) showed a weak stimulation of cAMP levels without increasing LOX, which we presume results from a driver of cAMP levels that is independent of osteogenic signaling. These findings support our hypothesis that insensitivity to GIP signaling occurs in diabetes in osteoblasts. Data confirm that amisulpride‐sensitive dopamine signaling is antagonistic against GIP signaling in osteoblasts, and that this is relevant to diabetic osteoblast biology.

### In vivo administration of amisulpride restores normal trabecular bone volume and structure

**Figure 6:**
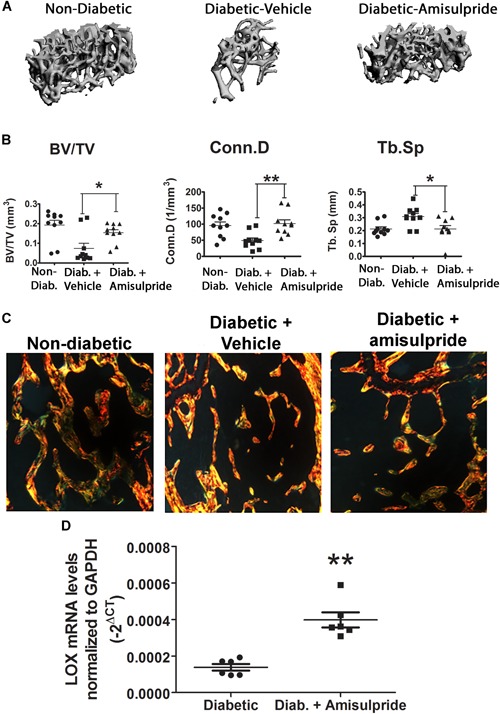
In vivo i.p. administration of the D2R inhibitor amisulpride restores a normal bone phenotype in diabetic mice. (*A*) µCT 3D tomographic reconstruction of femoral distal metaphysis in nondiabetic control, and diabetic vehicle‐injected or amisulpride‐injected mice. (*B*) Trabecular parameters. Displayed are those parameters that showed significance by one‐way ANOVA and Tukey's test for multiple comparisons, *n* = 10. (*C*) Recovery of collagen structure in diabetic mice after amisulpride treatment. Picrosirius red staining of femur trabeculae under polarized light, showing brightest images from diabetic bones. Representative images are shown, *n* = 3, with three sections per animal. (*D*) Partial recovery of tibia LOX mRNA levels in diabetic mice treated with amisulpride. Diabetic mice were maintained for 8 weeks to induce osteopenia, and then treated for 4 weeks with 10 mg/kg amisulpride or vehicle. RNA was directly isolated from tibia and assessed for LOX mRNA levels. Data are –2^ΔCt^, *n* = 6, **p < 0.01, by Student's *t* test with Welch's correction for unequal variance.

Amisulpride is approved in Europe as a treatment for schizophrenia because of its ability to potently and specifically inhibit D2R signaling, resulting in a decrease in dopamine signaling, and its effects have been well studied in humans and mice.^32^ Based on our in vitro studies, we hypothesized that in vivo administration of amisulpride at the therapeutic dosage of 10 mg/kg could reverse the diabetic bone phenotype seen in our mouse model. Mice were made diabetic and maintained for 8 weeks, permitting development of osteopenia. Mice were then treated with amisulpride or vehicle for an additional 4 weeks, after which bone structure was investigated by µCT analysis and compared to nondiabetic control and vehicle‐injected diabetic mice (*n* = 8). Results showed that diabetic mice treated with amisulpride significantly recovered trabecular bone structure, with the 3D tomogram of trabecular bone resembling that of the nondiabetic control mice. Quantitative analyses indicated restoration to nondiabetic control levels of the parameters BV/TV, Conn.D, and Tb.Sp (Fig. 6 *A*, *B*, *n* = 10, difference from diabetic *p* value < 0.05). Although diabetic mice remained hyperglycemic throughout the experiment (Supporting Fig. 5), these results show for the first time that interference with dopamine signaling in diabetes can restore normal bone health, pointing to D2R signaling inhibitors as providing new potential therapeutic strategies for diabetic bone disease.

### In vivo administration of amisulpride restores collagen organization and LOX mRNA levels

To examine if this restoration in the bone phenotype is accompanied by a rescue of the aberrant collagen structure in diabetic bone, Picrosirius red staining was performed on central sections from vehicle‐injected and amisulpride‐injected diabetic mice and compared to wild‐type control mice from the same bones already analyzed as in the previous paragraph by µCT. Results show that amisulpride‐treated diabetic mouse femur sections resemble that of the wild‐type in both the trabecular and cortical regions, with less bright yellow than the diabetic mice and a more mixed birefringence profile, indicative of an organized collagen structure and tightly packed collagen bundles, whereas vehicle‐injected diabetic mice maintain their less organized collagen structure (Fig. 6 *C*). Finally, RNA was isolated from the left tibias of diabetic vehicle‐injected and amisulpride‐injected mice and qRT‐PCR performed to examine levels of LOX. After amisulpride injections for 4 weeks we observed a threefold increase in the expression of LOX compared to vehicle‐injected diabetic mice (Fig. 6 *D*). However, nondiabetic mouse bones express on average about 10‐fold more LOX than amisulpride‐treated diabetic mice. These data, taken together with earlier work,^47^ suggest that a modest reversal of the dramatically downregulated LOX in diabetic mice could contribute to improved bone structure in diabetes. Further work to create relevant genetic mouse models are planned to further investigate the newly discovered relationships between diabetes, GIP, dopamine, LOX, and bone structure in diabetes.

## Discussion

Increased fracture risk due to diabetic bone disease is increasingly understood to be a burdensome health problem for both type I and type II diabetics. Not only are both type II and type I diabetes on the rise, but with advances in treatments for diabetes, diabetics are living longer lives.^48^ It is, therefore, important to develop new approaches to address osteopenia in light of the aging population of diabetic patients to ensure that mobility and quality of life are not further compromised. We therefore sought to identify a molecular mechanism for diabetic bone disease using a mouse model for type I diabetes. Studies of bone abnormalities in type I diabetes may have a degree of relevance to type II diabetes mellitus because both result in hyperglycemia and increased rates of hip and foot fractures that appear to be independent of BMD changes.^6^ In the type I mouse model, we show that diabetic bone has impaired collagen structure, severe trabecular defects, and decreased LOX RNA levels that appear to mediate diabetes’ effects on bone health. Our study also provides the first evidence of increased serum dopamine levels in diabetes, and further shows that dopamine inhibits GIP‐stimulated LOX expression in osteoblasts. Finally, treatment of diabetic osteopenic mice with therapeutic doses of amisulpride upregulated LOX levels by threefold, accompanied by restoration of collagen structure, and most importantly a near normal trabecular bone phenotype in diabetic mice.

Although LOX levels in amisulpride‐treated diabetic mice remained substantially below the mean level of wild‐type levels, trabecular bone structure was restored to near normal by amisulpride. Interestingly, an early study in copper‐deficient birds that exhibited osteolathyrism and low LOX activity, when supplemented with increasing levels of copper, indicated that bone LOX activity that is as low as 20% of normal control levels was sufficient to restore collagen crosslinking and bone strength.^47^ Here we have shown that LOX mRNA levels are reduced to nearly undetectable levels in bone and osteoblasts by diabetes, and that in vivo amisulpride supplementation to diabetic mice increases LOX levels by threefold. Taken together, these studies support the notion that the modest upregulation of LOX by amisulpride in diabetic mice can contribute to the observed restoration of bone structure under diabetic conditions.

Diabetic bone disease has unique characteristics that separate it from other bone disorders such as postmenopausal osteoporosis. Defects occur predominantly in the trabecular bone, which is more metabolically active than cortical bone, with a higher turnover rate and is typically more responsive to growth factors and hormones.^5^, ^49^ Conn.D, which is a trabecular bone–specific measure of the amount of solid connections in the trabecular meshwork,^33^ is significantly lower in diabetic mice, as well as Tb.Th, which measures the thickness of each unit of a trabecular connection.^33^ Importantly, diabetic mice show an increase in Tb.Sp, which is an average of the diameter of the spacing between the trabecula and is a critical determinant of trabecular bone quality. Poor trabecular bone quality correlates positively with an increase in fracture risk.^33^, ^50^, ^51^ The fact that the trabecular bone is more defective in diabetes therefore seems reasonable because trabecular bone is more likely to react negatively, at least in the short term, to a dysregulation of hormones and whole‐body homeostasis than the cortical bone. Interestingly, in a paper by Pachalis and colleagues,^11^ where BAPN, the pan‐LOX inhibitor, was used to induce osteolathyrism, defects in crosslinking were restricted to newly formed bone in the trabecula. This study also shows that although the mineral component of these bones was not affected, the mechanical performance of the whole bone was affected with decreases in maximum force to failure,^11^ a similar situation to that seen in diabetic bone.^52^ These findings point to the importance of biosynthetic collagen crosslinking to the functional integrity of bone strength.

Previous studies have reported that in diabetic bone the amount of collagen remains the same, but biosynthetic collagen crosslinking is decreased.^9^ Collagen crosslinking is known to have profound effects on collagen fibril organization and structure, with increased enzymatic crosslinking resulting in more tightly packed collagen fibrils.^9^ Fibril integrity may also dictate aspects of mineral deposition during bone formation that is thought to occur within the D‐periods of collagen fibrils.^53^ We reasoned that the Picrosirius red staining technique which is sensitive to collagen structure could be informative regarding the presence or absence of global structural organization defects in diabetic bone. Here we followed studies by Junquiera and colleagues,^36^ which supported that the brightness of the birefringence in Sirius red–stained bone sections corresponds to how much the stain penetrates the collagen fibers due to acid‐induced denaturation, and is, therefore, a measure of collagen fibril packing and maturity. As noted, our results show that, in diabetic mouse bone, there is a clearly different birefringence profile from that found in nondiabetic controls, including an abundance of bright yellow staining and collagen that appears striated. There is also an apparent loss of heterogeneity, with the collagen primarily appearing yellow and orange with very little green or red present. Thus, diabetic mice appear to have bone collagen that is less mature and more loosely packed, with loss of its higher‐order organization. Our results agree with previous studies that suggest diabetic bone disease results not from defects in the amount of matrix, but in the quality of the matrix and by association the quality of the bone.^9^

Although decreased enzymatic crosslinking has been shown in diabetic bone,^7^ and previous studies have shown decreased LOX protein in diabetic bone histologically,^52^ this is the first study that has examined LOX mRNA expression in diabetic bone. Based on previous studies that show LOX activity mediates collagen fibril diameter,^9^, ^19^, ^52^ we sought to determine if LOX expression was decreased in diabetic bone. Our results show a profound downregulation of LOX mRNA in diabetic bone, supporting our hypothesis that decreased LOX could be responsible for the decreased bone quality seen in diabetes.

GIP signaling is known to be dysregulated in diabetes, with tissues unable to respond to even supraphysiological doses of this hormone,^21^ and GIP is known to be normally anabolic to bone.^18^ Many mechanisms for the inhibition of GIP signaling in diabetes have been explored,^21^, ^54^ including desensitization of the GIPR with prolonged exposure to a ligand.^55^ Preliminary signaling studies (data not shown) did not suggest that a G‐protein receptor desensitization mechanism occurred in response to GIP in osteoblasts. We therefore explored a different mechanism. The anti‐incretin dopamine is able to inhibit glucose‐stimulated insulin secretion in pancreatic β‐cells by antagonizing GIP‐stimulated cAMP increases,^26^ and there is evidence that gut‐produced dopamine is responsible at least in part for the recovery from hyperglycemia seen in patients that have undergone bariatric surgery.^24^ However, this is the first study to our knowledge that shows in a diabetic mouse model that serum levels of dopamine are significantly increased compared to nondiabetic control animals. Therefore, the role of increased dopamine in the pathology of diabetes is now of great interest, and future experiments should examine peripheral dopamine levels in diabetic humans. At this time, we cannot exclude that amisulpride in the brain contributes to effects observed here. However, in this context, the use of other dopamine receptor inhibitors that more readily cross the blood‐brain barrier cause bone loss by interacting with dopamine receptors in the brain.^56^ This is opposite from effects of amisulpride provided here, further supporting that bone anabolic effects of amisulpride observed here are likely due to peripheral actions on osteoblasts, and not due to effects in the brain. It is important to understand that amisulpride only inefficiently crossed the blood‐brain barrier, resulting in a high ratio of peripheral to central nervous system concentrations.^57^

Data in vitro show that GIP upregulates predominantly LOX mRNA, and not other paralogs, and that LOX is the predominant expressed paralog in primary osteoblast cultures. Upregulation of LOX mRNA and protein was found to be dependent on adenylyl cyclase production of cAMP and PKA enzymatic activity. The receptor for GIP is a type II GPCR that acts through Gsα stimulatory G‐proteins to activate adenylyl cyclase and elevate cAMP levels.^41^ The Gsα pathway is important in human bone diseases,^42^ and anabolic treatments targeting this pathway are indicative of the crucial roles for cAMP in promoting bone formation, growth, and repair.^58^, ^59^ PKA activation is a consequence of cAMP elevation, and GIP stimulates insulin secretion in mouse pancreatic β‐cells through activation of PKA. Treatment of osteoblasts with dopamine inhibits GIP‐stimulated cAMP increases, and our data show that this pathway attenuates LOX expression in osteoblasts with increasing concentrations of dopamine.

Studies in primary osteoblasts derived from diabetic and nondiabetic mouse long bones showed that osteoblasts isolated from diabetic mice only express about 10% or less of the normal levels of LOX mRNA produced by nondiabetic osteoblasts, and very low levels of LOX protein. These findings agree with results showing the dramatic downregulation of LOX in RNA isolated directly from diabetic bone. The current report is the first to show that osteoblasts isolated from diabetic bone produce diminished LOX levels, consistent with the poor in vivo outcomes of bone quality and strength. The fact that osteoblasts derived from diabetic mice maintained their diabetic profile in culture is also an interesting observation. Typically, when primary cells are cultured they lose the characteristics they had in vivo^60^; however, the profound differences in LOX expression in osteoblasts isolated from diabetic mice were still present after 3 days in culture. Although this is a relatively short amount of time in culture, data may suggest the potential for epigenetic regulation of gene expression in diabetes in bone. Epigenetic mechanisms occur in diabetes, where hyperglycemia can cause permanent and heritable changes in gene expression profiles.^61^, ^62^

To confirm that decreased GIP signaling is specifically due to dopamine and not some other signaling dysregulation in diabetes, we rescued GIP signaling and LOX production in diabetic osteoblasts by inhibiting dopamine receptor activity. Dopamine has five receptors, all of which are expressed by osteoblasts.^27^ D2R in particular couples with Gαi/_o_ inhibitory G‐proteins that prevent adenylyl cyclase from making cAMP.^63^ Amisulpride is a specific D2R inhibitor, and has been shown to reverse dopamine inhibition of acetylcholine in the brain.^64^ It is classified as an atypical antipsychotic because of its potent and specific inhibition of D2R without effect on other receptors in the brain, and is commonly prescribed to treat schizophrenia and dysthymia in Europe, but not in the United States.^65^ Interestingly, primary cells isolated from diabetic mice were unable to respond to GIP with either increases in cAMP or increases in LOX expression. However, if diabetic osteoblasts were pretreated with amisulpride, GIP‐stimulated cAMP signaling and LOX expression recovered. These findings confirmed the specific role of dopamine in inhibiting GIP signaling in diabetic osteoblasts. Importantly, amisulpride by itself had no effect on LOX expression, indicating the ability of amisulpride to increase LOX expression is dependent on GIP.

To test whether amisulpride could restore bone health in vivo, we administered amisulpride via i.p. injections to diabetic osteopenic mice. Amisulpride restored the trabecular bone architecture almost back to that of nondiabetic control mice, with increases in BV/TV, decreases in Tb.Sp, and increases in Conn.D, which were the three parameters most affected in the initial µCT characterization of the bone from diabetic mice. There was also an apparent restoration in collagen architecture and of near‐normal LOX expression in the bones of the amisulpride‐injected diabetic mice. Data confirm the specific role of the organic matrix in diabetic bone disease, and provide evidence for the role of LOX in the pathology of diabetic bone disease.

Because amisulpride can act as an antipsychotic, we monitored our mice for changes in behavior and weight and observed no significant changes among the mice at the dosage used (Supporting Fig. 5). However, formal behavioral analysis may be needed in the future if related agents are to be considered to be used to treat diabetic bone disease in humans. Amisulpride has been reported to increase prolactin levels in treated patients, which could be an undesired side effect of this particular drug.^66^ However, D2R undergoes alternative splicing resulting in two different transcripts,^67^ and there is the possibility that pharmacologic targeting of different isoforms of D2Rs could avoid some of the reported negative side effects. Amisulpride has also been shown to restore insulin secretion and lower blood glucose levels in diabetic mice,^32^ and deregulation of glucose homeostasis is a common feature in patients taking antipsychotics.^68^ Modest antidiabetic effects of amisulpride have been reported.^69^ We considered the possibility that recovery of a normal bone phenotype in our diabetic mice was simply due to a recovery of insulin secretion and reversal of hyperglycemia. To control for this, we performed insulin ELISAs on our amisulpride‐injected diabetic mice and compared results to nondiabetic and diabetic vehicle‐injected mice. Insulin levels were slightly increased in our amisulpride‐injected mice, but not significantly when compared to the vehicle‐injected diabetic mice, and were still significantly lower than the nondiabetic control mice (Supporting Fig. 6). Furthermore, blood glucose readings, although slightly decreased in our amisulpride‐injected mice, remained in the diabetic range throughout the course of the study (Supporting Fig. 5). In addition, the recovery we are seeing in the bone phenotype in amisulpride‐treated diabetic mice was dramatic compared to untreated diabetic controls, and cannot be explained by the modest decreases we are seeing in blood glucose. Because of our careful monitoring of the mice and attempts to control for dopaminergic effects in other parts of the body, we conclude that the positive effect of dopamine inhibition on bone in diabetes is mostly likely due to a recovery in GIP signaling in osteoblasts.

**Figure 7:**
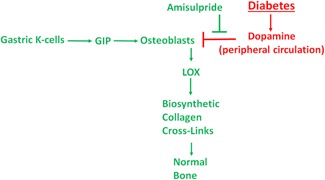
Proposed relationships between diabetes, dopamine, osteoblasts, and bone structure supported by data presented. Red text and symbols refer to diabetes and dopamine effects that inhibit osteoblast function, while green text and symbols refer to normal osteogenic processes and the observed positive effect of amisulpride.

In conclusion, data provide evidence for the specific critical role of the LOX paralog regulation in diabetic bone disease. This study also further shows how important gut‐derived incretins and anti‐incretins are to bone health, independent of their effects on other tissues. Furthermore, our research suggests that peripheral dopamine has effects on bone, and is dysregulated in diabetes. Taken together, our findings that dopamine inhibits strong GIP‐stimulated LOX production in osteoblasts, combined with significantly elevated serum levels of dopamine observed in the diabetic mice, and partial restoration of LOX levels and near full recovery of trabecular bone structure by amisulpride, point to the conclusion that elevated gut‐derived dopamine in diabetes is a major mechanism of diabetic bone disease. This report defines a novel molecular mechanism for diabetic bone disease and highlights a complex interconnectedness of the skeleton with the pancreas and the gut. A summary of a working model of the relationships supported by the data presented is found in Fig. 7.

## Disclosures

PCT has served as a consultant for Pharmaxis Corportaion, Frenchs Forest, NSW, Australia. All other authors declare no potential conflicts of interest.

## Supporting information

Supporting information.
