# Supplementary material for: Impaired Gastric Hormone Regulation of Osteoblasts and Lysyl Oxidase Drives Bone Disease in Diabetes Mellitus
Source: JBMR Plus. 2019 Aug 7;3(10):e10212. doi: 10.1002/jbm4.10212 (PMC6820454; doi:10.1002/jbm4.10212)
Supplement: Supplementary file 1 — Supporting information. [file JBM4-3-na-s001.docx]

**
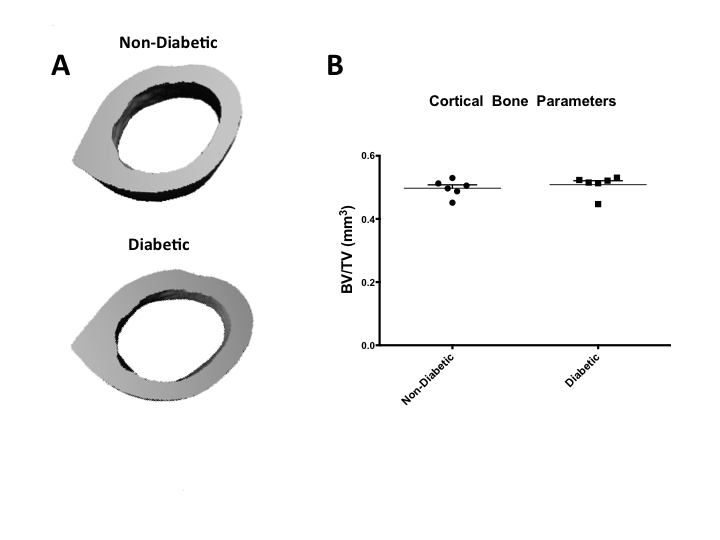
**

**Figure S1.** **Cortical bone volume/total volume from distal metaphysis of the femur in non-diabetic and diabetic mice.** One-way ANOVA and Tukey’s test for multiple comparisons was employed for analysis, n=6.

**
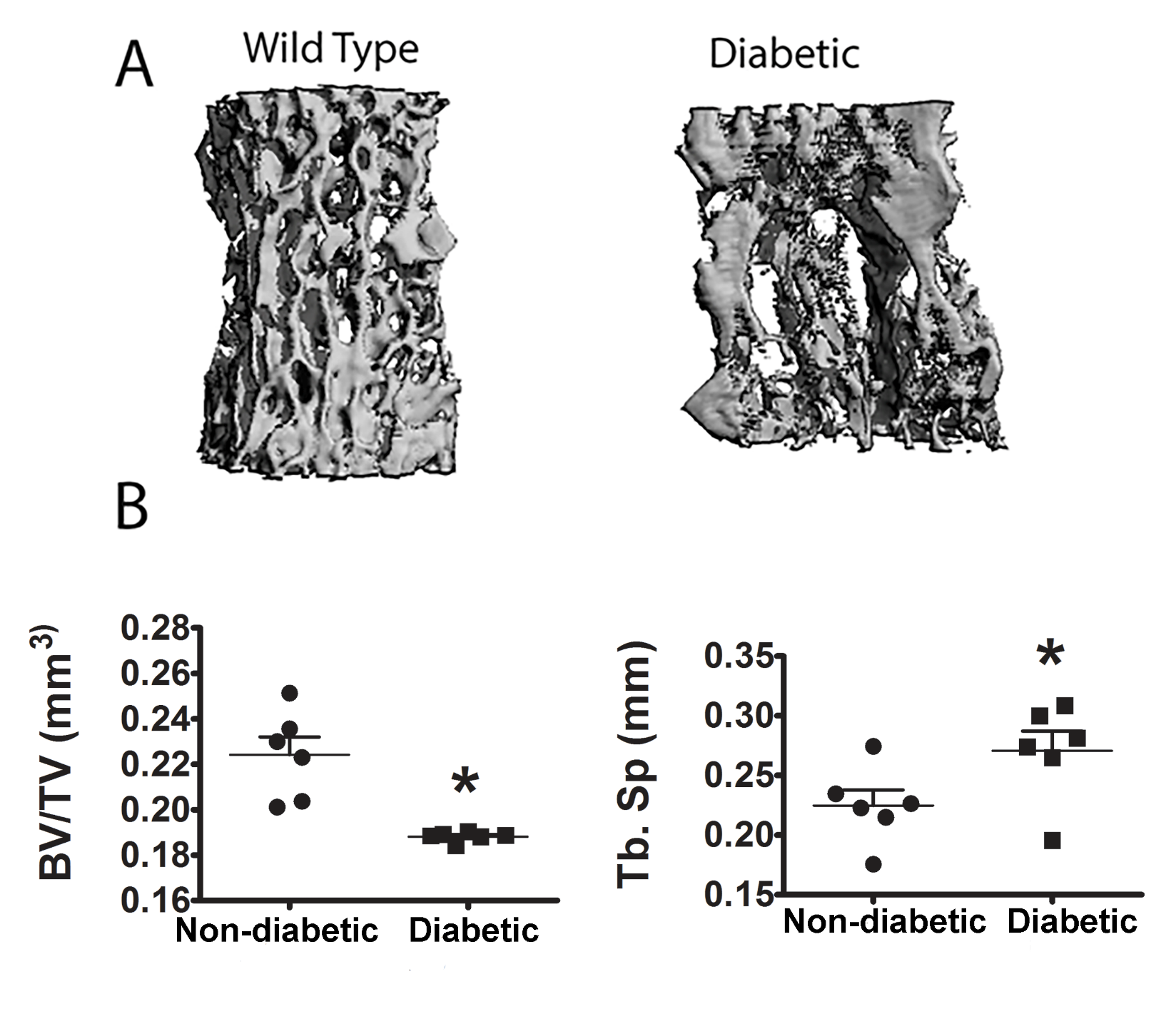
**

**Figure S2. L5 vertebrae parameters of nondiabetic and diabetic mice.** Micro-CT 3-D tomogram (A) and trabecular bone parameters (B) from vertebral trabecular compartment in non-diabetic and diabetic mice. *p<0.05; n=6. Only parameters that showed significance are shown.

**
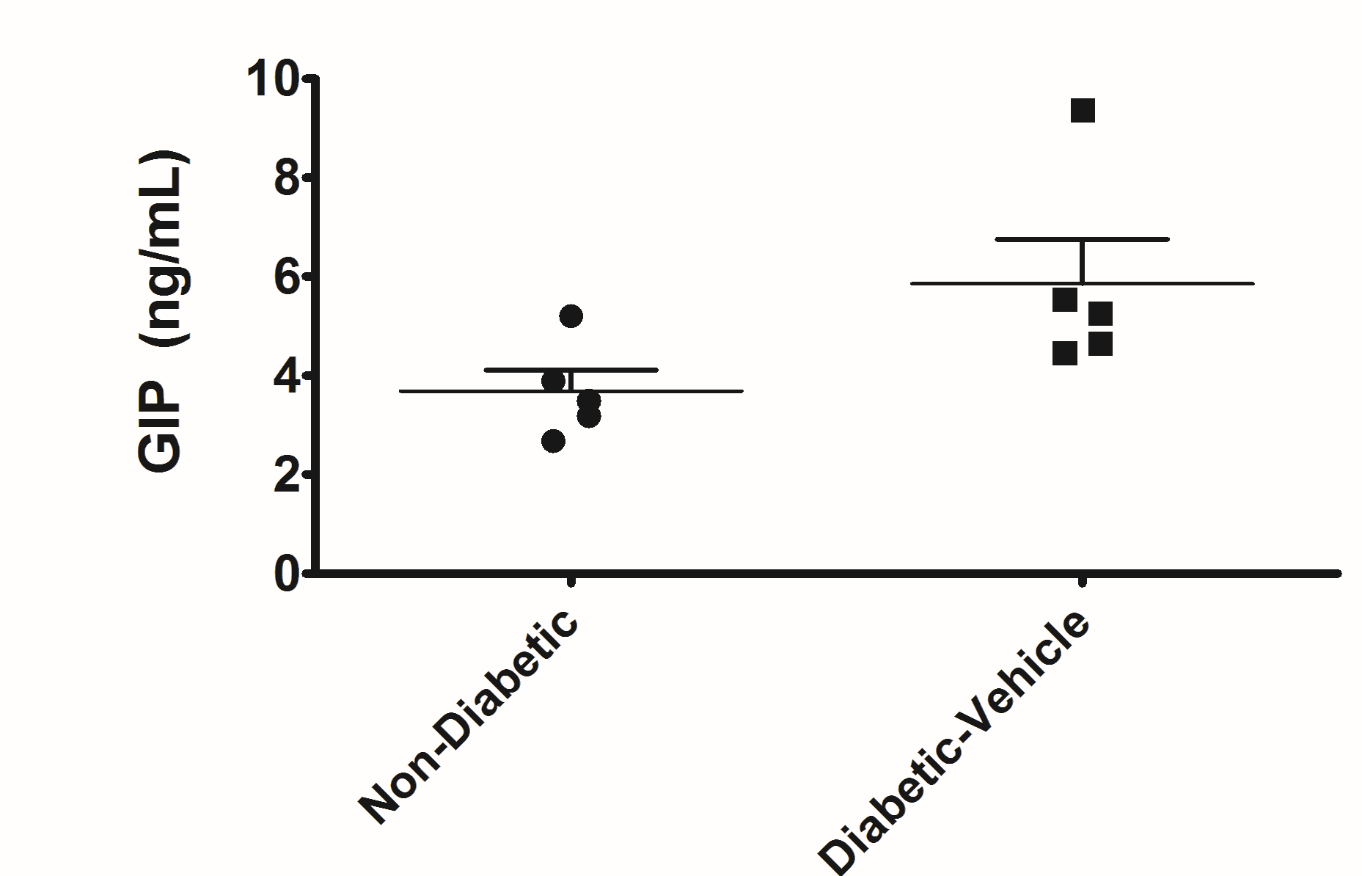
**

**Figure S3. Diabetic mice do not have decreased GIP levels.** Serum was isolated from blood taken from diabetic and vehicle control mice. Data are means +/- SD, p>0.05, by student’s t-test, n=5 per group.

**
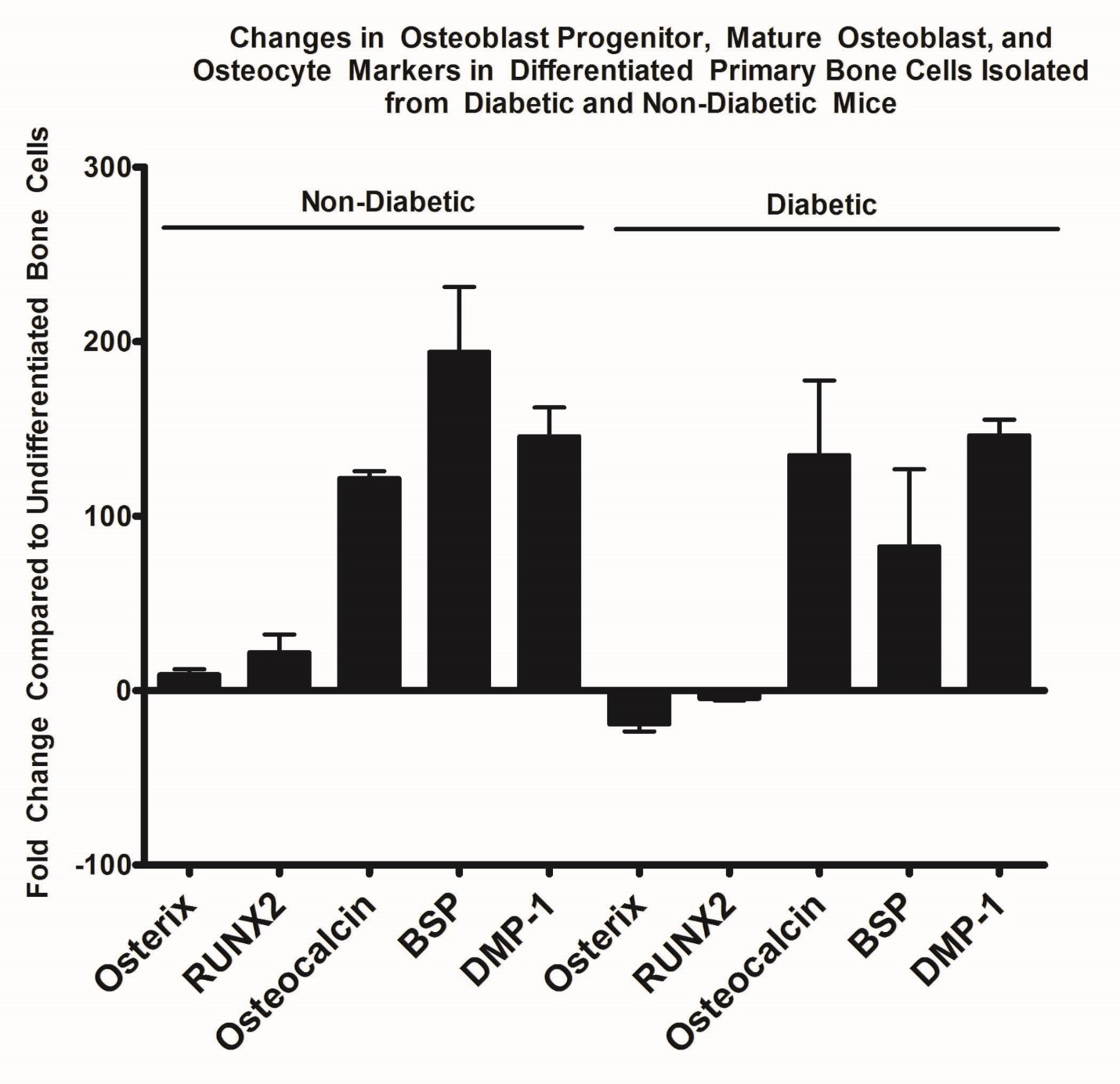
**

**Figure S4. qRT-PCR for osteoblast mRNA markers.** Primary bone explant cells were incubated in osteogenic differentiation medium (100 nM dexamethasone, 0.05 mM L-ascorbic acid, 10 mM β-glycerophosphate) for 3 weeks and medium changed every 3 days. RNA was isolated after three weeks. Data represent fold change in reference to RNA isolated from primary bone cells at day 4. Data were calculated using the 2 ^-(ΔΔCT)^ method using GAPDH reference gene, n=5 per group.

**
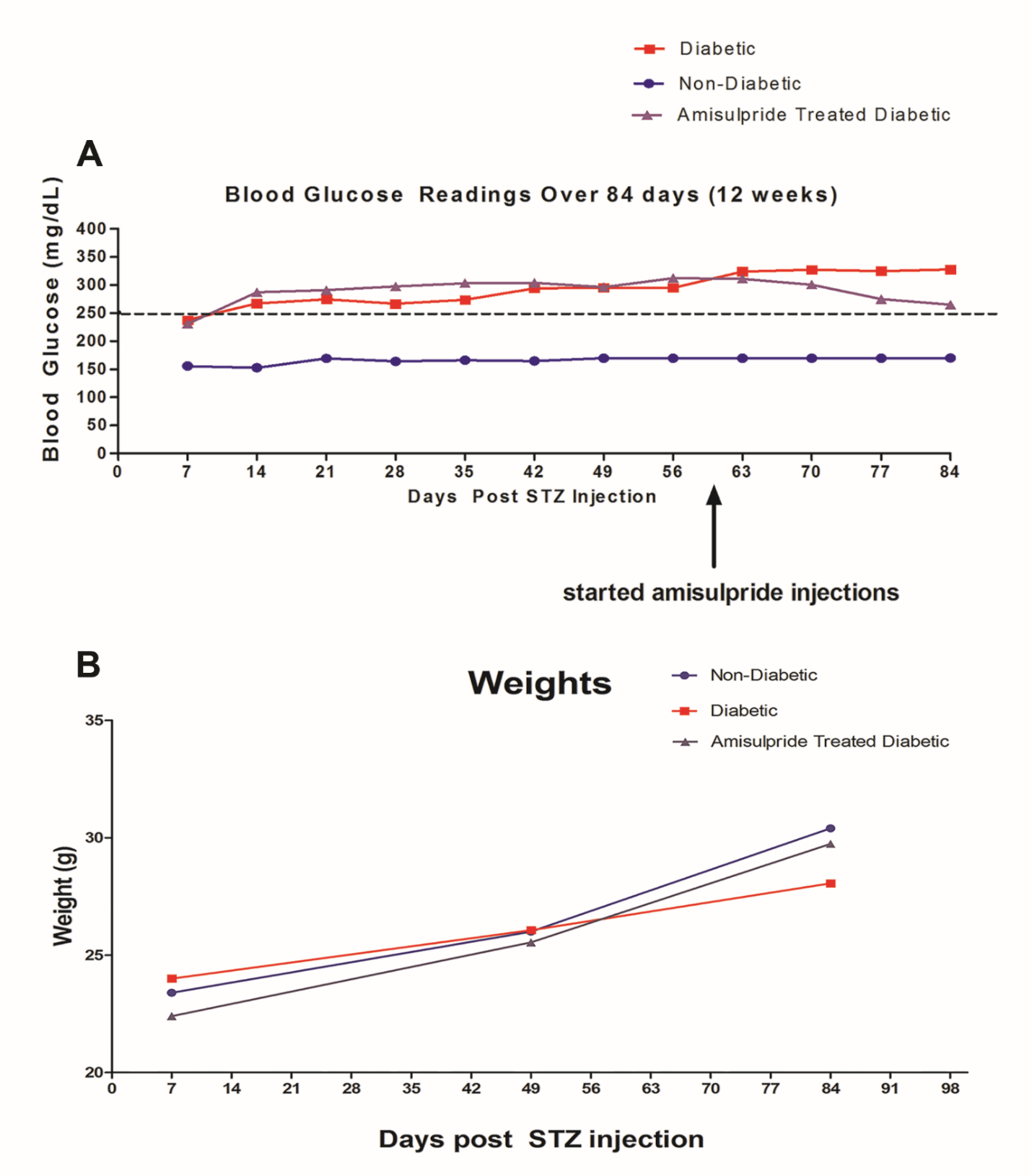
**

**Figure S5. Serum glucose (A) and mouse weights (B) of nondiabetic, diabetic and amisulpride-treated mice as a function of time.** Times indicated are days after initiation of the 5 daily single ip injections of 40 mg/kg streptozotocin**.**

**
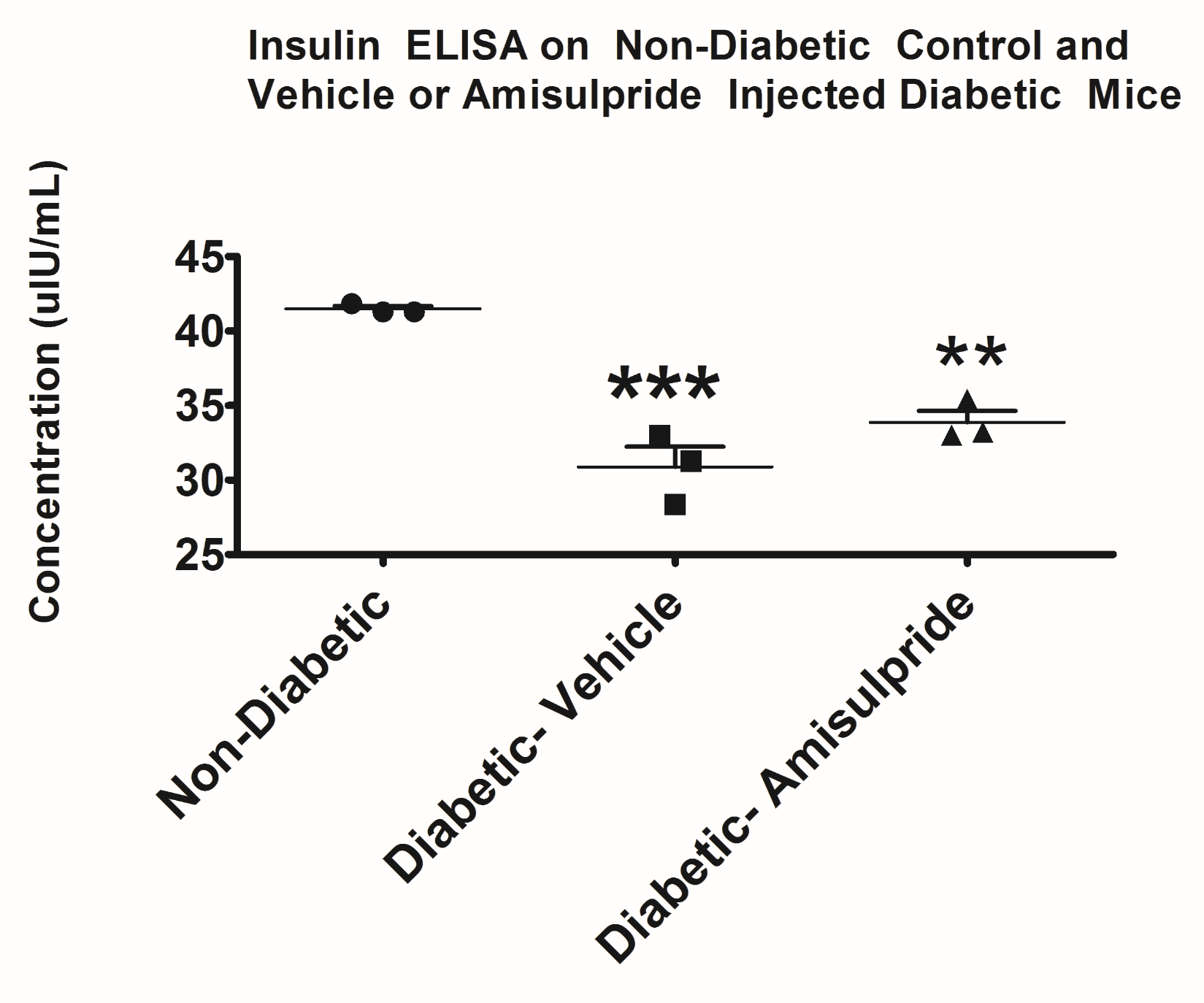
**

**Figure S6. Amisulpride injected diabetic mice do not have increased serum insulin levels.** Serum was isolated from blood taken from non-diabetic control, and amisulpride or vehicle injected diabetic mice. Data are means +/- SD, **p<0.01, ***p<0.001 by student’s T-test vs non-diabetic control, n=3 per group.
